# Detection of antimicrobial impact on gram-negative bacterial cell envelope based on single-cell imaging by scanning electron microscopy

**DOI:** 10.1038/s41598-023-38198-3

**Published:** 2023-07-12

**Authors:** Akiko Hisada, Erino Matsumoto, Ryo Hirano, Mami Konomi, Jacques Yaacoub Bou Khalil, Didier Raoult, Yusuke Ominami

**Affiliations:** 1 Healthcare Innovation Center, Research and Development Group, Hitachi, Ltd., 1-280, Higashi-Koigakubo, Kokubunji-shi, Tokyo 185-8601 Japan; 2 Core Technology and Solutions Group, Hitachi High-Tech Corporation, Tokyo, 105-6409 Japan; 3 Institut Hospitalo-Universitaire Méditerranée Infection, 13005 Marseille, France; 4 Consulting Infection Marseille, 13008 Marseille, France

**Keywords:** Antimicrobial resistance, Scanning electron microscopy

## Abstract

Rapid determination of drug efficacy against bacterial pathogens is needed to detect potentially resistant bacteria and allow for more rational use of antimicrobials. As an indicator of the antimicrobial effect for rapid detection, we found changes in image brightness in antimicrobial-affected bacteria by scanning electron microscopy (SEM). The cell envelopes of unaffected bacteria were stained with phosphotungstic acid (PTA), whereas the entire cells of affected bacteria were stained. Since tungsten density increases backscattered electron intensity, brighter bacterial images indicate lethal damage. We propose a simplified method for determining antimicrobial efficacy by detecting damage that occurs immediately after drug administration using tabletop SEM. This method enabled the visualization of microscopic deformations while distinguishing bacterial-cell-envelope damage on gram-negative bacteria due to image-brightness change. *Escherichia coli*, *Acinetobacter baumannii*, *Enterobacter cloacae*, *Klebsiella pneumoniae*, and *Pseudomonas aeruginosa* were exposed to imipenem and colistin, which affect the cell envelope through different mechanisms. Classification of single-cell images based on brightness was quantified for approximately 500 bacteria per sample, and the bright images predominated within 5 to 60 min of antimicrobial treatment, depending on the species. Using intracellular PTA staining and characteristic deformations as indicators, it was possible to determine the efficacy of antimicrobials in causing bacterial-cell-envelope damage.

## Introduction

Antimicrobial resistance (AMR), in which bacterial changes render antimicrobials ineffective, is a major public health threat, and a comprehensive assessment revealed that bacterial AMR is a leading cause of death worldwide^1^.

Rapid identification of AMR is essential for appropriate treatment and prevention of the spread of AMR. However, the gold standard method of antimicrobial susceptibility testing (AST), which is based on culturing isolated bacteria, requires approximately 3 days from blood sample collection to determine resistance to a candidate drug^2^. To reduce testing time, novel methods of rapid AST are now in clinical practice and their clinical impact is being investigated^3^. Newer phenotypic AST methods by various approaches to detect growth inhibition in shorter periods have been reported^4^. To determine the susceptibility of bacteria with these AST methods, culturing time is required until the amount or suspension concentration of proliferated bacteria exceeds the technical detection limit.

The AST based on single-cell analysis now offers the potential for more rapid testing by monitoring the growth rate of individual bacteria in microfluidic channels^5, 6^, analyzing the motion pattern of individual cells^7^, and quantifying damaged bacteria by fluorescence-labeled flow cytometry^8, 9^. This is because the phenomenon caused by antimicrobials can be detected with a small number of bacteria, thus eliminating a long incubation period. The AST based on microscopy of antimicrobial-induced morphological changes at the single-cell level is promising^4^. The advantage of microscopy is that it can capture the characteristic morphological changes in susceptible bacteria immediately after drug administration, such as filamentation, swelling, bulging, and lysis, corresponding to the mechanism of action^10^, which enables detection of drug effects without prolonged culturing. Therefore, a microscopic approach to rapid AST was carried out by combining analysis of morphological changes at the single-cell level and short-time growth measurements^11^.

However, bacterial population growth can be detected with the gold standard AST methods using general indicators such as colony formation and suspension turbidity regardless of bacterial species and antimicrobials, whereas the microscopic approach is complicated by the need to characterize which morphological changes result in bacterial growth inhibition for each combination of antimicrobial action and target molecules on bacterial species. It is also difficult to distinguish between lethal and non-lethal deformations.

We developed a simple microscopic single-cell analysis method that involves using tabletop SEM to reveal the effect of antimicrobials on the bacterial cell envelope by combining characteristic bacterial deformation related to the antimicrobial action and changes in image brightness derived from PTA staining as image indicators. Technically, we found that PTA staining can be used to detect lethal damage to the bacterial cell envelope. Based on this finding, we analyzed the effects of the antimicrobials, imipenem, one of the carbapenems and colistin, one of the drugs effective against carbapenem-resistance on five clinically problematic gram-negative bacilli species identified as priority pathogens of AMR by World Health Organization^12^.

## Results

### Detection of bacterial-cell-envelope damage using PTA staining

To detect antimicrobial effects on the bacterial cell envelope under SEM, we chose PTA staining because we compared backscattered electron (BSE) images of bacteria using five stains, PTA without pH adjustment, neutralized PTA, ammonium molybdate, sodium tungstate, and TI blue (Nissin EM), that are easier to handle than uranium acetate and lead, which are commonly used for electron microscopy staining, and found that a 10% PTA aqueous solution without pH adjustment more brightly stained bacteria treated with colistin than untreated bacteria (Fig. S1). The pH of the solution was below 2.0. Assuming that low pH PTA staining can be used to detect damaged bacteria, 10% PTA aqueous solution was used in the following experiments.

**Figure 1:**
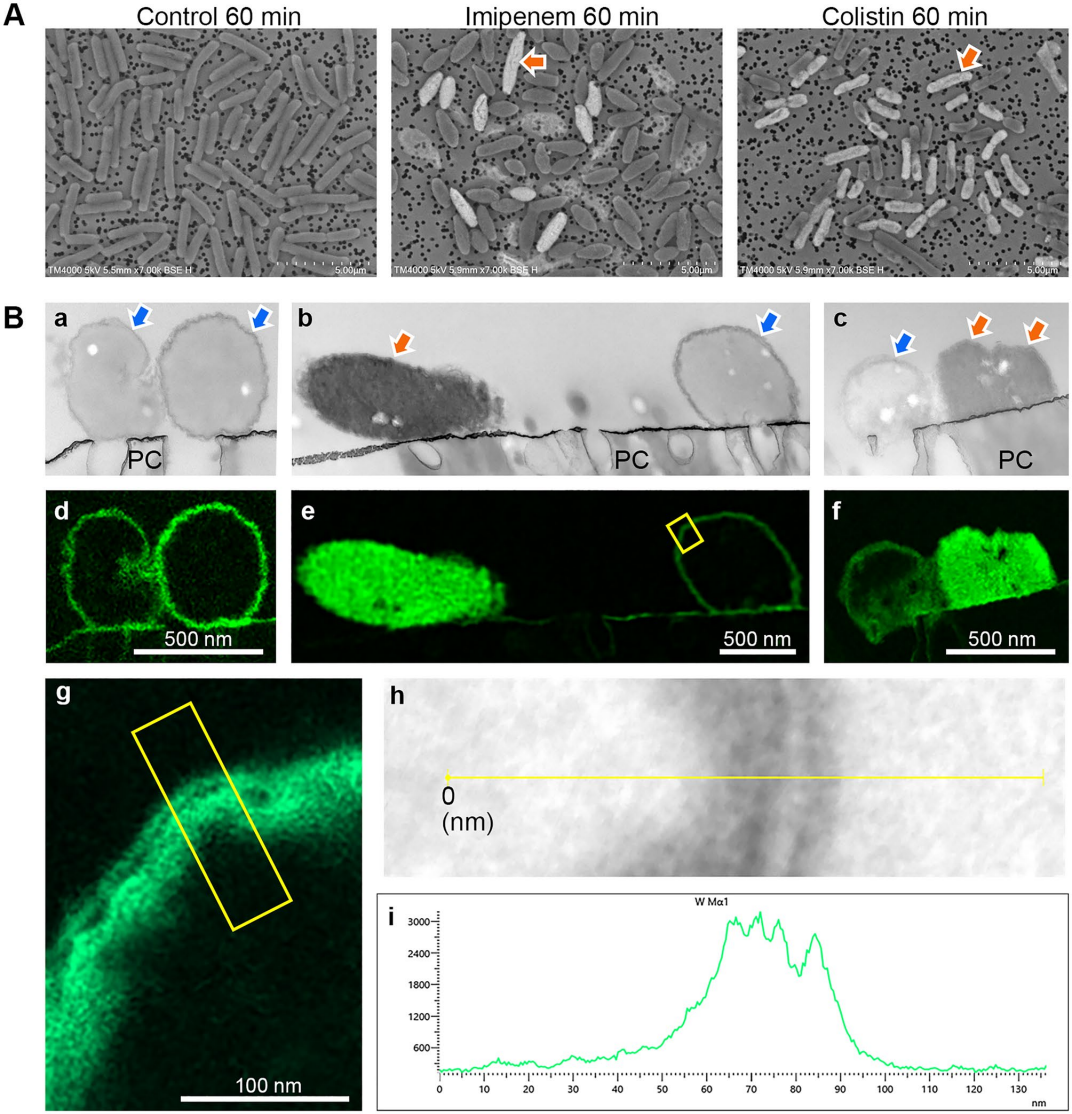
Intracellular staining with PTA in antimicrobial treatment. (**A**) *Escherichia. coli* (ATCC 25922) was cultured in M–H medium (control) or M–H medium containing imipenem or colistin for 60 min, collected on an iso-pore polycarbonate membrane. BSE images were captured by tabletop SEM before resin embedding. Red arrows indicate examples of brightly stained bacteria. (**B**) (a, b, c) Transmission electron images of ultrathin sections prepared from samples in A by BF-STEM, and (d, e, f) tungsten mapping (green) of a, b, c, respectively by EDX. a and d, control; b and e, imipenem treated; c and f, colistin treated. PC, polycarbonate membrane. Bacteria with high electron density in the surface (blue arrows), and bacteria with high electron density throughout (red arrows). (g) Magnified image of tungsten mapping in the rectangular region in e. (h) BF-STEM image of line scan position in the rectangular region in g, (i) signal intensity profile of tungsten by line scan indicated with yellow line in h, 0 nm on the horizontal axis corresponds to the position of 0 in h.

To confirm intracellular staining for differences in image brightness of bacteria with and without antimicrobial treatment, *Escherichia coli* (ATCC 25922) susceptible to both imipenem and colistin were prepared on an iso-pore membrane, stained with 10% PTA aqueous solution, and BSE images were taken under SEM (Fig. 1A), showing low-bright bacteria in the untreated sample (Fig. 1A, control) and a mixture of low-bright bacteria and bright bacteria in the antimicrobial treated for 60 min (Fig. 1A, imipenem and colistin). Intracellular staining was examined by bright field-scanning transmission electron microscopy (BF-STEM) and energy dispersive X-ray spectroscopy (EDX) of ultra-thin sections of the same samples (Fig. 1B). In the transmission electron image of the control sample, the electron density was higher on the surface layer of the bacterial section (Fig. 1B a). In contrast, the images of the antimicrobial-treated samples showed a mixture of bacteria with high electron density in the surface layer and bacteria with high electron density throughout (Fig. 1B b,c). Elemental analysis confirmed that the high electron density was due to PTA staining, consistent with the tungsten mapping (Fig. 1B d,e,f). Magnified tungsten mapping of the surface-stained bacteria revealed a layer of low tungsten between regions of high tungsten (Fig. 1B g), which was confirmed by line-scan intensity profiles (Fig. 1B h,i), suggesting that those bacterial surface structures are components of the cell envelope^13^. Since the intensity of BSE is higher for the density of heavier elements, the difference between low-bright bacteria and bright bacteria under SEM was attributed to the difference in subcellular staining with PTA; low-bright BSE image, in which the cell envelope was stained, and bright BSE image in which the entire cell was stained. This suggests that such striking stainability changes during imipenem or colistin treatment reflect lethal damage to the bacterial cell envelope on the basis of the antimicrobial mechanism of action.

### Single-cell image classification

**Figure 2:**
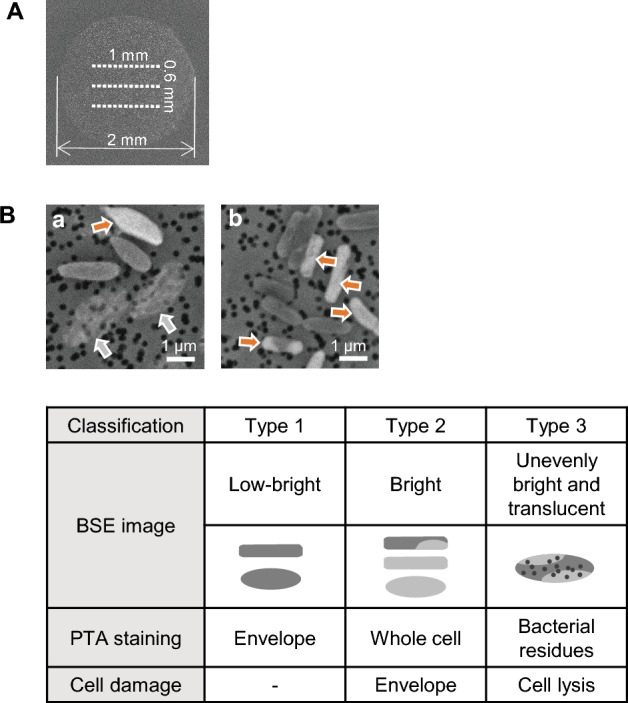
Single-cell image classification. (**A**) Bacterial sample was collected uniformly in a circular area 2 mm in diameter of the iso-pore membrane. 13 images × 3 row (positions indicated with white dotted lines) were automatically acquired at a magnification of 7000× using SEM. (**B**) Magnified BSE images of *E. coli* (ATCC 25922); a, imipenem treated; b, colistin treated. Red arrows indicate type 2, grey arrows indicate type 3, and others are classified as type 1.

We developed a method for determining the antimicrobial efficacy on bacterial strains on the basis of single-cell image brightness derived from PTA staining. The initial bacterial concentration in the antimicrobial treatment was adjusted at 10^5^ CFU/ml orders of magnitude, the same as with the broth dilution assays of the gold standard AST method. After antimicrobial treatment, to disperse bacteria on an iso-pore membrane at an average density of 10–20 bacteria per field of view (18 × 14 µm) of a 7000 × SEM image, 0.5 ml of bacterial suspension was collected in a 2 mm-diameter circular area on the iso-pore membrane (Fig. 2A).

An acceleration voltage of 5 kV was selected as the SEM condition for single-cell imaging, which did not allow the background membrane-pore images to show through when BSE images of relatively small intact *Pseudomonas aeruginosa* were acquired (Fig. S2). Thirty-nine images (13 images × 3 rows) were automatically acquired in the bacteria-collection area (Fig. 2A). Images with bacterial residues after cell lysis were included in the analysis, even if there was no obvious bacterial outline. After excluding images of background membrane without bacteria and those containing non-bacterial debris, the first 25 images per sample were subjected to image classification and quantitative analysis, and excess images were not included in the analysis. Since an average of 10–20 single-cell images were captured per SEM image, we classified approximately 250–500 bacteria in 25 images per sample.

Single-cell images of bacteria were manually classified into three types by visual inspection on the basis of the brightness of the image, regardless of swelling or other deformation (Fig. 2B). Type 1 was a low-bright bacteria image due to cell-envelope staining, type 2 was a bright bacteria image due to whole-cell staining. Partially bright bacteria were classified into type 2. Type 3 was an unevenly bright image of bacteria due to a mixture of images of bacterial components of the same brightness as type 2 and translucent images of a background membrane with pores showing through due to leakage of intracellular components. Images with residual bacteria without obvious bacterial outlines were included in Type 3.

It was possible to distinguish type 1 from types 2 by normalizing the image brightness and contrast settings using the brightness of the polycarbonate of the membrane and that of the pores, even among different samples (Fig. S3). The brightness distribution of type 1 partially overlaps that of the polycarbonate substrate, while the brightness distribution of types 2 is separated from that of the polycarbonate substrate and type 1.

The percentage of each type of bacteria to the total number of bacteria per sample was determined. For type 3, in samples where individual bacteria could no longer be identified due to lysis, the number of type 3 bacteria was calculated by subtracting the number of types 1 and 2 bacteria from the average of the total number of bacteria at the earlier sampling time. The time course from the onset of initial cell envelope damage, classified as type 2, to cell lysis, classified as type 3, was analyzed.

### Kinetics of antimicrobial-induced bacterial-cell-envelope damage

**Figure 3:**
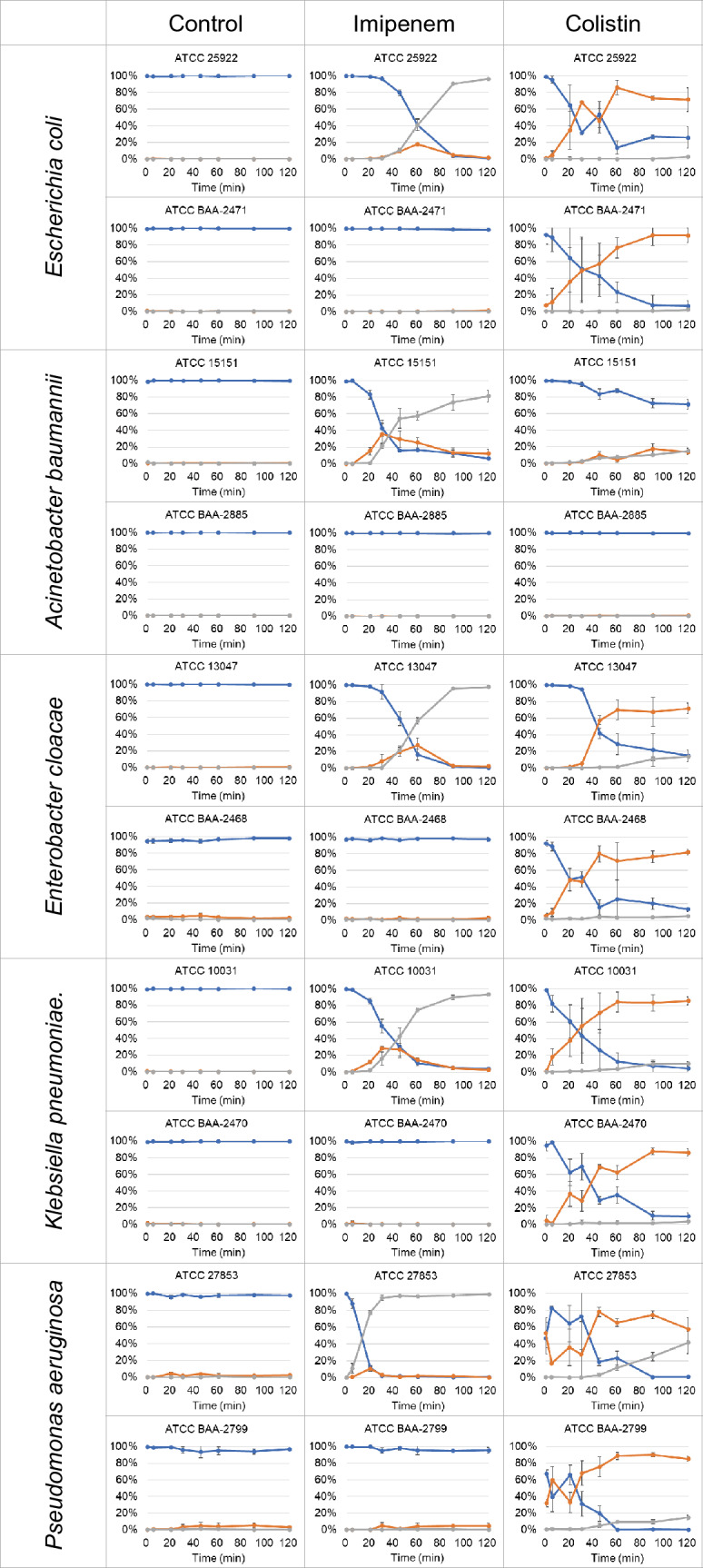
Time course of the percentage of single-cell images of bacteria classified into three types. Antimicrobial treatment (control, imipenem, colistin) were conducted simultaneously in parallel and the percentage of each type of bacteria to the total bacterial count was determined. Means and standard deviations of the three independent experiments are shown; type 1 of low-bright bacteria (blue), type 2 of bright bacteria (red), type 3 of bright and translucent bacteria (gray); vertical axis: percentage of bacteria (%), horizontal axis: incubation time with antimicrobials (min).

Single-cell image classification based on image brightness was carried out on bacterial strains of *Escherichia coli*, *Acinetobacter baumannii*, *Enterobacter cloacae*, *Klebsiella pneumoniae*, and *Pseudomonas aeruginosa* (Table 1). Bacterial suspensions were sampled at 0, 5, 20, 30, 45, 60, 90, and 120 min from the start of imipenem and colistin exposure at the EUCAST breakpoint concentration that discriminates between susceptibility and resistance^14^ and fixed with glutaraldehyde for time-resolved analysis of antibacterial effect (Fig. 3).

**Table 1:** Bacterial strains.

Species	Strain	Antimicrobials	MIC (mg/L)^a^	Susceptibility^b^
*Escherichia coli*	ATCC 25922	Imipenem	0.125	S
		Colistin	0.125	S
	ATCC BAA-2471	Imipenem	> 32	R
		Colistin	0.125	S
*Acinetobacter baumannii*	ATCC 15151	Imipenem	0.94	S
		Colistin	0.38	S
	ATCC BAA-2885	Imipenem	> 32	R
		Colistin	8	R
*Enterobacter cloacae*	ATCC 13047	Imipenem	0.38	S
		Colistin	0.5	S
	ATCC BAA-2468	Imipenem	> 32	R
		Colistin	0.38	S
*Klebsiella pneumoniae*	ATCC 10031	Imipenem	0.19	S
		Colistin	0.5	S
	ATCC BAA-2470	Imipenem	> 32	R
		Colistin	0.38	S
*Pseudomonas aeruginosa*	ATCC 27853	Imipenem	1.5	S
		Colistin	2	S
	ATCC BAA-2799	Imipenem	> 32	R
		Colistin	2	S

^a^E-test was conducted to confirm the minimal inhibitory concentration (MIC).

^b^S, MIC lower than breakpoint concentration; R, MIC higher than breakpoint concentration.

No obvious decrease in type 1 percentage was observed in the control samples by 120 min (Fig. 3 control). With imipenem treatment (Fig. 3 imipenem), in five imipenem-susceptible strains of *Escherichia coli* (ATCC 25922), *Acinetobacter baumannii* (ATCC 15151), *Enterobacter cloacae* (ATCC 13047), *Klebsiella pneumoniae* (ATCC 10031), and *Pseudomonas aeruginosa* (ATCC 27853), the percentage of type 2 bacteria began to increase temporarily from 5 to 30 min after dosing then decreased. Type 3 bacteria appeared almost simultaneously with type 2 bacteria and continued to increase, reaching nearly 100% by 120 min, suggesting that damage to the cell envelope resulting from inhibition of cell wall synthesis by imipenem^15^ occurs in 5 to 30 min and quickly leads to lysis. The timing of the decrease in type 1 bacteria to less than 50% was 20 min for ATCC 27853, 30 min for ATCC 15151, 45 min for ATCC 10031, and 60 min for ATCC 25922 and ATCC 13047. In contrast, no significant damage was evident in the imipenem-resistant strains (Fig. 3), *Escherichia coli* (ATCC BAA-2471), *Acinetobacter baumannii* (ATCC BAA-2885), *Enterobacter cloacae* (ATCC BAA-2468), *Klebsiella pneumoniae* (ATCC BAA-2470), and *Pseudomonas aeruginosa* (ATCC BAA-2799). Inter-experimental differences were small, with standard deviations ranging from ± 0 to ± 13%, depending on the incubation time, in three independent experiments for each strain, indicating that the onset of morphological damage caused by imipenem in the isolates used in this study varies in time for individual bacteria but occurs over a nearly constant time course for the population as a clonal strain when treatment conditions are the same.

With colistin treatment (Fig. 3 colistin), in colistin-susceptible strains of *Pseudomonas aeruginosa* (ATCC 27853, ATCC BAA-2799), about half the samples at 0 min were type 2, indicating that damage occurs during the few minutes between sampling and fixation. In *Escherichia coli* (ATCC 25922, ATCC BAA-2471)*, Enterobacter cloacae* (ATCC 13047, ATCC BAA-2468)*,* and *Klebsiella pneumoniae* (ATCC 10031, ATCC BAA-2470), the percentage of type 2 bacteria began to increase from 5 to 30 min after dosing. Type 2 bacteria increased to around 80% from 45 to 60 min and retained by 120 min. In ATCC 27853, the percentage of type 3 bacteria increased to 40% by 120 min, suggesting that damage to the cell envelope resulting from the detergent effect of colistin on the cell membranes^16^ occurs from a few minutes then progresses and that the progression of lysis is slower than with imipenem. The timing of the decrease in type 1 bacteria to less than 50% was 30 to 45 min for these eight colistin-susceptible strains. In *Acinetobacter baumannii* (ATCC 15151), types 2 and 3 gradually increased to about 15% by 90 min, 30% by 120 min, with the proportion of type 1 remaining higher than in susceptible strains of other species. Additional data indicate that changes over time in the total number of bacteria compared to controls suggest that colistin inhibited the bacterial growth (Fig. S4), indicating that ATCC 15151 was susceptible. In contrast, no significant damage was evident in the colistin-resistant strain *Acinetobacter baumannii* (ATCC BAA-2885) and colistin treatment did not inhibit the bacterial growth (Fig. S4).

In the colistin treatment, there were inter-experimental differences, with standard deviations ranging from ± 0 to ± 39%, depending on the incubation time, in three independent experiments for each strain, especially in the early timing of the treatment. After about 45 min, the difference among the experiments decreased.

An epsilometer test (E-test) was conducted to confirm the antimicrobial susceptibility of the strains (Table 1), and there was no apparent change in susceptibility in three independent experiments. The minimal inhibitory concentration (MIC) of each imipenem-susceptible strain of the five species ranged from 1/16 to 1/2 of the EUCAST breakpoint^14^, and that of each of the colistin-susceptible strains ranged from 1/16 to the same value of the EUCAST breakpoint, so bacterial growth was not detected at the breakpoint concentration. The MIC of each imipenem-resistant strain was 10 times higher, and the colistin-resistant strain was 4 times higher than the EUCAST breakpoint; every resistant strain proliferated at the breakpoint concentration.

In summary, this method was used to analyze the onset of antimicrobial effects in a bacterial population over time on a single-cell basis and discriminate the antimicrobial susceptibility of bacterial strains to imipenem and colistin, which affect the bacterial cell envelope by different mechanisms of action.

### Bacterial morphology

**Figure 4:**
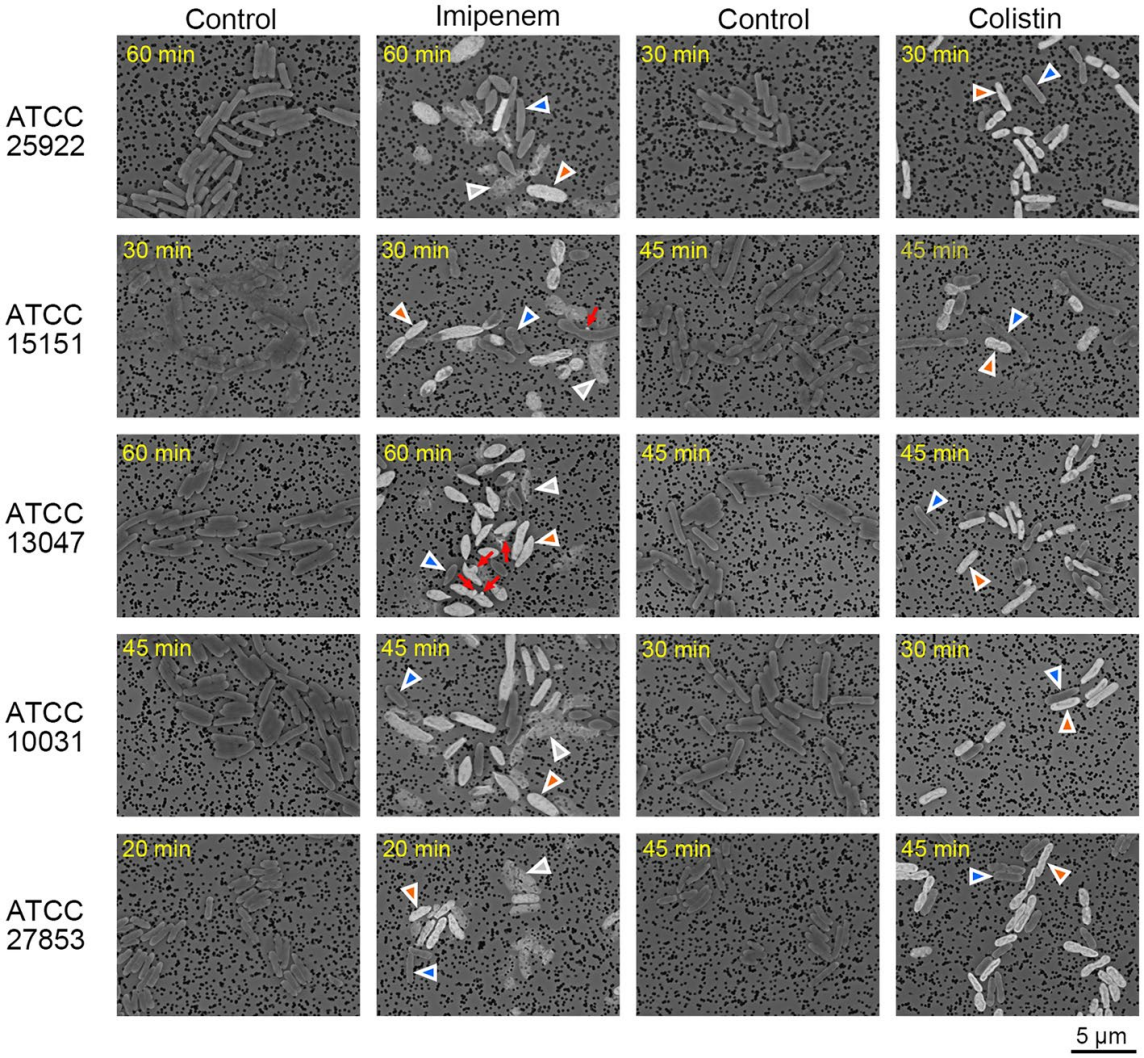
Deformation caused by antimicrobial action in five gram-negative bacilli species, *Escherichia coli* (ATCC 25922), *Acinetobacter baumannii* (ATCC 15151), *Enterobacter cloacae* (ATCC 13047), *Klebsiella pneumoniae* (ATCC 10031), *Pseudomonas aeruginosa* (ATCC 27853). Bacterial images when type 1 of low-bright was reduced to approximately 50% or less after the start of imipenem or colistin administration; for colistin-treated ATCC 15151 at the time of type 1 decrease (see Fig. 3). Arrowheads indicate examples of bacteria classified as type 1 (blue), type 2 (red), and type 3 (gray). Red arrows indicate bulging.

Bacterial images of the susceptible strains treated with imipenem or colistin when type 1 became less than 50% (see Fig. 3) are shown in Fig. 4. A mixture of types 1, 2, and 3 bacteria were observed in the microcolonies of the imipenem-treated samples, and types 1 and 2 bacteria were observed in the colistin-treated samples, while the bacteria in the controls collected at the same time were type 1. The imipenem-treated samples showed normal shape, swelling, and bulging in both types 1 and 2 staining. The colistin-treated samples, however, did not show significant deformation at this timing. Type 2 bacteria swelled slightly in ATCC 27853 and shrunk slightly in ATCC 13047 and ATCC 10031. In ATCC 15151, the shorter bacteria tended to show type 2 staining, and the longer bacteria of type 1 staining were relatively dominant. Although there may be a difference from the authentic bacteria size due to artifacts from sample preparation, we were able to reveal lethal damage while capturing characteristic deformations caused by the inhibition of cell-wall synthesis^10^ and damage on the membrane^16^. The advantage of using electron microscopy is that when quantifying lethal damage based on the brightness of individual bacteria, the observation of microscopic bacterial deformation in the same image allows confirmation that the change in brightness is due to a specific antimicrobial effect.

## Discussion

We found that in bacteria unaffected by antimicrobials, the bacterial cell envelope is stained with 10% PTA aqueous solution, whereas in affected bacteria the entire cell is stained (Fig. 1). It has been reported that PTA has an affinity for polysaccharide components and stains the cell-surface coat in resin sections^17^. Polysaccharides are one of the major components of the bacterial cell envelope^18^, and the localization of polysaccharides in the bacterial cell wall has been demonstrated by transmission electron microscopy (TEM) of ultrathin sections stained with low pH PTA^19^. In this study, we confirmed the subcellular localization of tungsten by EDX in sections of bacteria stained with PTA before embedding and sectioning (Fig. 1B). In the section of intact cells, tungsten was localized on the bacterial surface, indicating that low pH PTA has an affinity for the cell wall even by cell staining. In the section of antimicrobial-affected cells, tungsten was uniformly distributed throughout the cell. Intracellular staining remained after rinsing, suggesting that PTA adsorbs to intracellular components, but the mechanism of uniform staining needs to be investigated. The change in stainability was thought to indicate a loss of inner membrane integrity due to imipenem inhibiting cell-wall synthesis or colistin damaging the cell membrane, which has been observed morphologically in ultrathin sections^20, 21^. TEM examination of cell-membrane damage is a skilled and time-consuming process, whereas SEM examination of the whole cell with PTA staining is a straightforward process. We developed a simple method on the basis of single-cell imaging to detect antimicrobial effects in terms of lethal damage due to loss of inner membrane integrity.

SEM can be used to visualize bacterial morphology with high resolution; tabletop SEM images were used to measure bacterial size changes caused by imipenem^22^. In this study, we designed a multi-well filtering unit (Fig. S5) and developed a preparation procedure with a brief fixation and PTA staining to compare multiple samples quantitatively and reproducibly. It was possible to capture antimicrobial-induced morphological changes in 15 min of sample processing without long fixation time, critical point drying, and metal deposition that are required in conventional SEM sample preparation. By obtaining more accurate morphological information using tabletop SEM, which is as simple as routine optical microscopy, antimicrobial efficacy can be evaluated with a small number of bacteria immediately after drug treatment. Based on the validation of the principle in this report, the system has potential for use in routine inspections by automating the process from sample preparation to image analysis.

To address the challenge that setting morphological image indicators for each combination of antimicrobials and bacterial species complicates the rapid detection of antimicrobial efficacy by microscopy, we proposed two image indicators to detect the effects of antimicrobials in causing cell-envelope damage. The first is the BSE image brightness derived from PTA staining (Fig. 2), which identifies lethal damage to the cell envelope, as a general indicator (Fig. 3). The second is the characteristic deformation of the bacteria in accordance with the mechanism of action of the antimicrobials as a specific indicator, which is revealed in the bacteria morphologically preserved in glutaraldehyde (Fig. 4). We demonstrated that in a combination of two antimicrobial agents, imipenem and colistin, and five gram-negative bacilli, it was possible to discriminate between susceptible and resistant strains within one hour of incubation. AST based on single-cell image analysis using optical microscopy has also been conducted. Since deformation by antimicrobial action is not always lethal and observed even in resistant bacteria, multiple indicators have been combined to determine susceptibility by monitoring the growth of resistant bacteria or disappearance due to lysis of susceptible bacteria for hours^11, 23^. Our method can detect the occurrence of initial damage due to antimicrobial effect with our two image indicators, which may contribute to the rapid detection of resistant bacteria.

In this study, quantitative results have been generated from brightness-based data, as morphology criteria, such as swelling, has not been taken into account in the image classification (Figs. 2 and 3). It has been reported that resistant bacteria can be detected by quantifying imipenem-induced swelling in SEM images^22^. Although swelling is not a sufficient sole indicator to determine susceptibility for beta-lactam agents, as it also occurs in resistant bacteria^11^, it allows direct quantification of the effect of antimicrobials acting on the cell wall. The measurement of deformation may allow the relationship between antimicrobials and the expression levels of bacterial receptors or resistance mechanisms to be verified. As our method allows deformations and lethal states to be observed in the same image, it may be possible to develop new susceptibility criteria by quantifying and relating both indicators.

For further evaluation of our method, it will be necessary to compare resistant bacteria with lower MIC and resistant bacteria with different resistance mechanisms. As shown in the data on susceptible strains in this report, there are individual differences in the time at which antimicrobial damage occurs, suggesting that there is a distribution of susceptibility at the individual level even within the same strain; as the time differences were reproduced in three experiments, we assume that there is a strain-specific distribution under certain culture conditions. If the antimicrobial breakpoint concentration is within the range of this distribution of lower MIC resistant isolates, then some individuals are considered to be lysed and the remaining individuals will grow and be confirmed as resistant. Therefore, it is hypothesized that intermediate types of resistance can be determined by the proportion of individuals that grow under antimicrobial treatment. It is assumed that the detection of resistant individuals is influenced by the resistance mechanism and its level of expression and an analysis that takes this into account is required.

PTA-staining properties in the cell envelope of gram-positive bacteria and the detection of bacterial damage due to different antimicrobial actions will also be investigated. We think that intracellular staining by PTA was mainly due to loss of cell membrane integrity. Therefore, if other mechanisms of action, such as inhibition of DNA or protein synthesis, result in a lethal bacterial state and loss of cell membrane integrity, it is speculated that antimicrobial efficacy can be detected as the brightly stained bacterial image by PTA staining. However, it is considered difficult to detect the initial onset of antimicrobial activity as in the case of direct cell envelope damage.

Because bacterial morphology is affected by various factors such as the genetic background of the cells, culture environment, and growth stage^24, 25^, it was thought that these factors may affect antimicrobial susceptibility. In *Acinetobacter baumannii* (ATCC 15151) exposed to colistin, for example, classification showed a gradual increase in damaged bacteria, with a total of about 30% of types 2 and 3 at 120 min (Fig. 3), and morphological observations tended to show a type 2 staining in the shorter bacteria and a relative predominance of the longer type 1 (Fig. 4). It has been reported that Acinetobacter are short rods in the logarithmic phase of growth but often become more coccoid in the stationary phase^26^. In this study, the short bacilli tended to be more damaged than the long bacilli, suggesting that the growth phase may be related to the onset of initial damage caused by colistin^27^, but further study is needed.

Because our method visualizes the phenotype of individual cells in response to antimicrobial effects in a time-resolved manner, it is possible to quantitatively confirm the growth of a heterogeneously resistant population^28^ by calculating the percentage of individuals showing normal morphology at a given time, even if most bacteria are lethally damaged. Our method also visualizes diverse morphological changes that reflect the mechanism of action as well as lethality, which may be useful in the search for substances that affect bacteria.

In conclusion, we found that lethal damage to the bacterial cell envelope can be detected by changes in the brightness of BSE images under SEM because antimicrobial exposure alters the permeability of PTA into the bacterial cell, causing intracellular staining. On the basis of this finding, we developed a simple method to detect the antimicrobial susceptibility of bacterial strains immediately after drug treatment by image classification of individual bacteria.

## Materials and methods

### Bacterial strains

*Escherichia coli* (ATCC 25922, ATCC BAA-2471), *Acinetobacter baumannii* (ATCC 15151, ATCC BAA-2885), *Enterobacter cloacae* (ATCC 13047, ATCC BAA-2468), *Klebsiella pneumoniae* (ATCC 10031, ATCC BAA-2470) and *Pseudomonas aeruginosa* (ATCC 27853, ATCC BAA-2799) are from American Type Culture Collection (ATCC).

### Culture conditions

Frozen stocks of the bacterial strain were grown on Mueller–Hinton broth (M–H) agar medium, and selected colonies were grown overnight on M–H liquid medium, diluted at a concentration of 0.5 McFarland, and subcultured for approximately 4 h then used for antimicrobial treatment during the log growth phase. For imipenem-resistant strains (ATCC BAA-2471, ATCC BAA-2468, ATCC BAA-2470), M–H medium containing 25 mg/L was used for pre-culture according to ATCC instructions. To confirm the MIC of the strains for each experiment, a portion of the pre-cultured bacterial suspension was used for E-test (bioMérieux).

### Antimicrobial treatment

Sensi-Disc (imipenem, colistin, BD) were added to M–H liquid medium to prepare a medium containing antimicrobial at twice the concentration of the breakpoint by EUCAST^14^ and filtered before use for the following treatments. The bacterial suspension was prepared in M–H liquid medium at a concentration of 2 McFarland and adjusted to the order of 10^5^ CFU/ml by diluting 5 × then twice 10 × dilutions. Bacteria were exposed to antimicrobials by mixing the bacterial suspension and M–H liquid medium containing twice the concentration of antimicrobials in a 1:1 ratio. Final antimicrobial concentrations were set at 2 mg/L imipenem and 2 mg/L colistin for *E. coli*, *Acinetobacter baumannii*, *Enterobacter cloacae*, and *Klebsiella pneumoniae*, and 4 mg/L imipenem and 2 mg/L colistin for *Pseudomonas aeruginosa*. Bacterial suspensions were slowly agitated at 37 °C and sampled over time. Immediately prior to sampling, bacteria in the antimicrobial treatment solution were suspended by pipetting.

### SEM sample preparation and image capturing

A polycarbonate hydrophilic iso-pore track-etched membrane filter with a thickness of 25 µm, pore diameter of 0.2 µm, and pore density of 5 × 10^8^/cm^2^ was purchased (ipPORE, it4ip). The membrane surface was coated with platinum palladium for conductivity and placed in an originally designed multi micro filtration unit with a 20-well removable funnel (Fig. S5A). Bacterial suspensions without (−) or with (+) an antimicrobial agent were agitated in an incubator (Fig. S5B), sampled over time, injected into the funnel of the filtration unit (Fig. S5C). For the following sample preparation procedure, liquid was poured into the wells of funnel and retained on the membrane, and negative pressure was applied under the membrane when the liquid was removed (Fig. S5C). Bacteria were washed with 0.85% saline, fixed with 2.5% glutaraldehyde in saline for 5 min, flushed with pure water tree times, stained in 10% phosphotungstic acid aqueous solution for 2 min, and flushed with pure water three times. Bacteria were collected in a 2 mm-diameter circular area on the membrane (Fig. S5C). The entire procedure takes about 15 min to process one membrane using the filtration unit. There was a time lag of about 3 min between sampling the bacteria and fixing them with glutaraldehyde because of the duration for aspiration.

The membrane with bacteria collected on the surface was taken out of the unit and air-dried. The membrane was placed in the sample chamber of a tabletop scanning electron microscope (TM4000 plus, Hitachi High-Tech) and vacuumed. BSE images were automatically captured at a magnification of 7000×.

### Elemental analysis in ultra-thin sections

*Escherichia coli* (ATCC 25922) treated with imipenem or colistin for 60 min were collected on the membrane, fixed with glutaraldehyde and stained with PTA as described above; to collect bacteria at high density, the volume of bacterial suspension per well of the funnel (Fig. S5) was 1.5 ml for control and 2.0 ml for imipenem and colistin. Bacteria collected on the iso-pore membrane were air dried and examined under SEM prior to resin embedding, then immersed in t-butanol and finally embedded in epoxy resin. Ultrathin Sections 60 nm thick and perpendicular to the membrane were prepared and placed on a neoprene coated copper grid. Sections were examined under a scanning electron microscope (SU9000, Hitachi High-Tech), BF-STEM images were captured at an acceleration voltage of 30 kV, and tungsten elemental maps and line profile were acquired using an EDX detector (Ultim Max TLE, Oxford instruments).

## Supplementary Information

Supplementary Information.

## Data Availability

All data is included in the manuscript and supporting information.
