# Supplementary material for: Detection of antimicrobial impact on gram-negative bacterial cell envelope based on single-cell imaging by scanning electron microscopy
Source: Sci Rep. 2023 Jul 12;13:11258. doi: 10.1038/s41598-023-38198-3 (PMC10338463; doi:10.1038/s41598-023-38198-3)
Supplement: Supplementary file 1 — Supplementary Information. [file 41598_2023_38198_MOESM1_ESM.pdf]

Supplementary Information for

Detection of antimicrobial impact on Gram-negative bacterial cell envelope based on single-cell imaging by scanning electron microscopy.

Akiko Hisada [1*], Erino Matsumoto [1], Ryo Hirano [2], Mami Konomi [2], Jacques Yaacoub Bou Khalil [3], Didier Raoult [4] and Yusuke Ominami [2]

[1] Healthcare Innovation Center, Research & Development Group, Hitachi, Ltd., Tokyo, 185-8601, Japan

[2] Core Technology & Solutions Group, Hitachi High-Tech Corporation, Tokyo, 105-6409, Japan

[3] Institut Hospitalo-Universitaire Méditerranée Infection, Marseille, 13005, France

[4] Consulting Infection Marseille, Marseille, 13008, France

* Corresponding author

Akiko Hisada

Email: akiko.hisada.uf@hitachi.com

This PDF file includes:

Figures S1 to S5

SI Reference

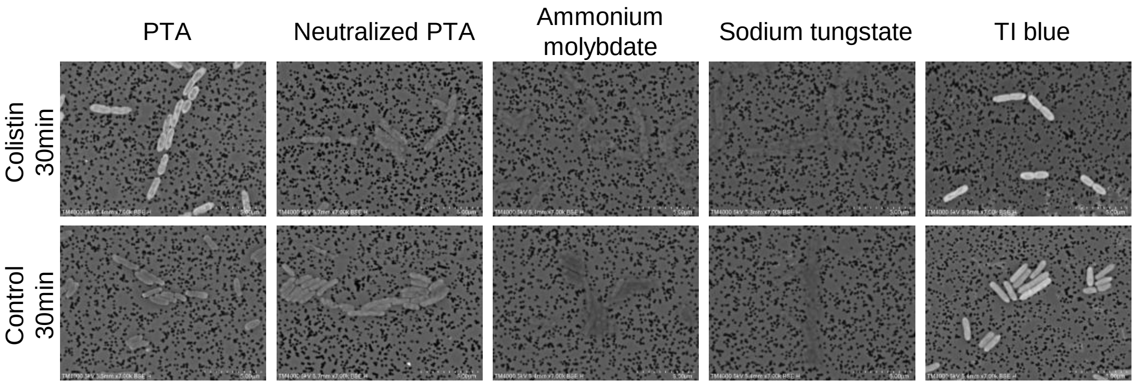

Fig. S1. Comparison of stain.

Colistin-treated (Colistin) and untreated (Control) susceptible *Pseudomonas aeruginosa* were fixed in 2.5% glutaraldehyde and stained with five different staining solutions. Backscattered electron (BSE) images were taken under scanning electron microscopy (SEM) at an acceleration voltage of 5 kV. 10% phosphotungstic acid (PTA) aqueous solution stained colistin-treated bacteria more strongly than controls. When other stains were used, there was no obvious difference in staining intensity between colistin-treated and untreated bacteria; 3% neutralized PTA weakly stained both, 10% ammonium molybdate solution or 10% sodium tungstate solution stained neither, and TI blue (Nissin EM) strongly stained both.

When PTA concentrations of 0.25, 1, 2, and 10% were tested, all showed strong staining in bacteria treated with colistin, and the 10% aqueous solution was selected because of its reproducibility among the experiments.

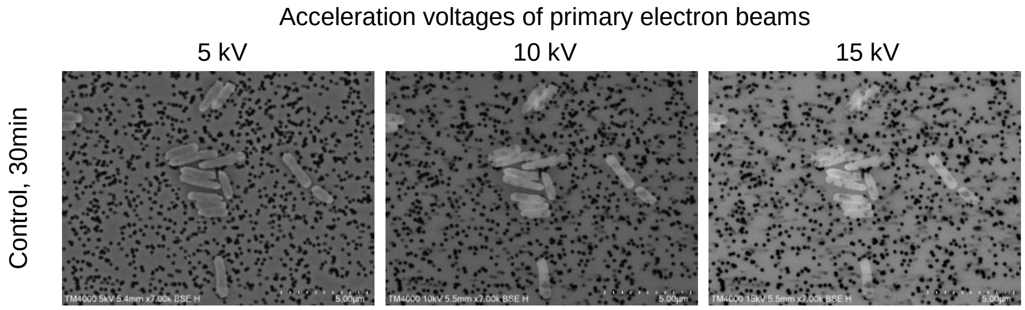

Fig. S2. Comparison of acceleration voltages of primary electron beams in SEM.

BSE images of *Pseudomonas aeruginosa* fixed with 2.5% glutaraldehyde and stained with 10% PTA aqueous solution were acquired at different SEM acceleration voltages: at 5 kV, the bacterial morphology was visualized, but primary electron beams emitted at 10 and 15 kV penetrated the bacteria and the pattern of pores in the membrane substrate overlapped the bacterial image.

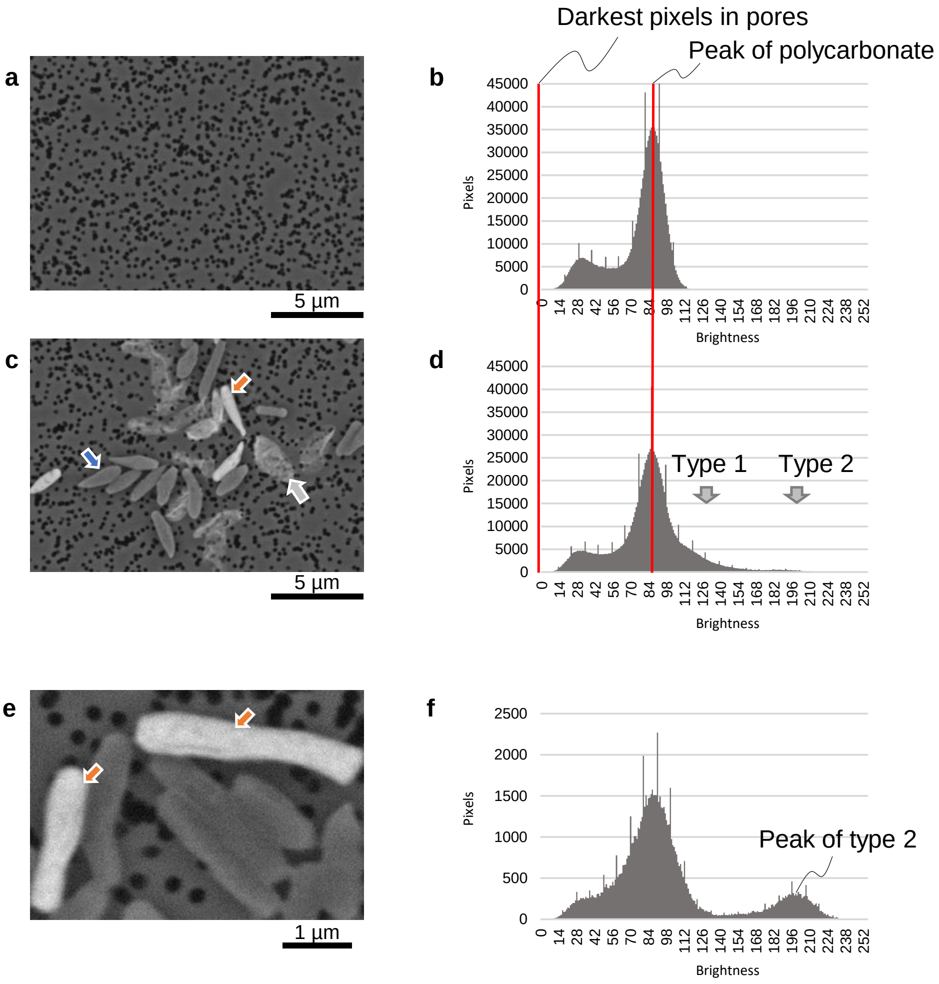

Fig. S3. BSE images and corresponding brightness histogram.

Brightness and contrast in images can be normalized on the basis of the brightness of the peak of the polycarbonate membrane and darkest pixels of the pores. (a) (b) Iso-pore membrane; (c) (d) *E. coli* treated with imipenem for 60 min; (e) (f) types 1 and 2 bacteria. Arrows indicate examples of type 1 (blue), type 2 (red), and type 3 (gray) in (c) and (e). Type 2 can be distinguished from type 1 and polycarbonate by brightness threshold 140 (f). Histograms were plotted using ImageJ [1].

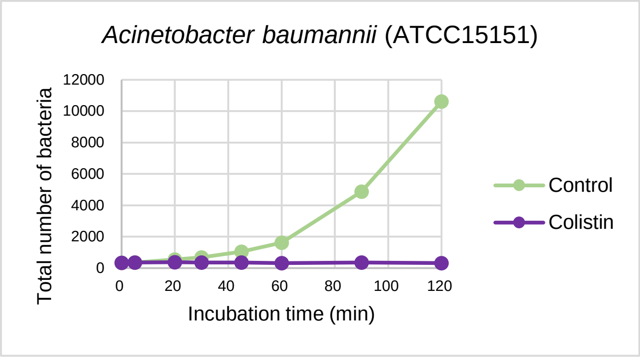

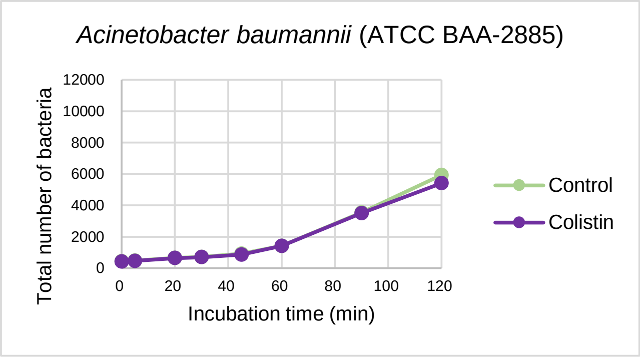

Fig. S4. Time course of bacterial counts during colistin (-/+) incubation.

Total number of bacteria in 25 images per sample in a single experiment. This trend was confirmed in three independent experiments. The susceptible strain (ATCC 15151) grew more than 30-fold within 2 hours of incubation without antimicrobial treatment (Control), but not with colistin treatment (Colistin). In contrast, the resistant strain (ATCC BAA-2885) grew about 14-fold with or without colistin.

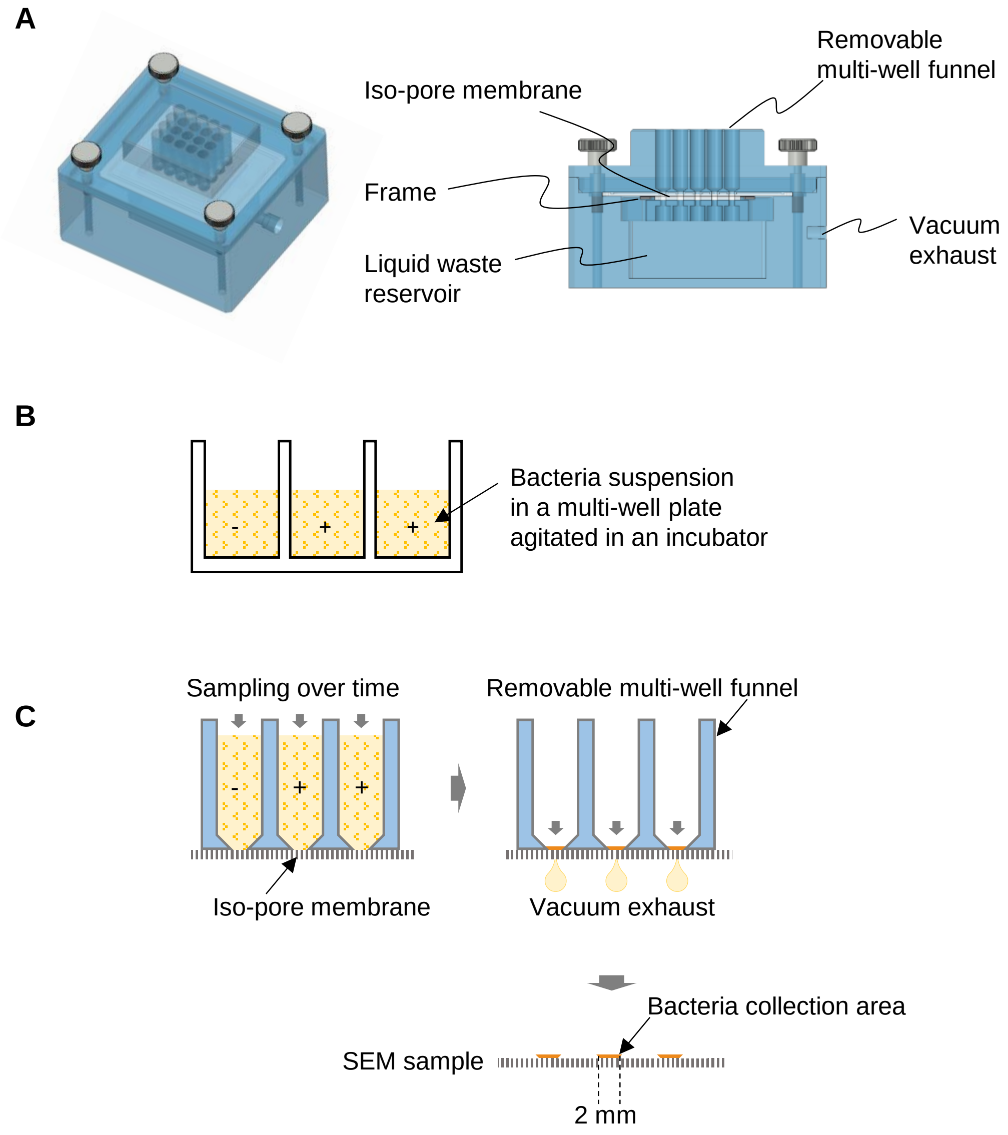

Fig. S5. Multi micro filtration unit.

(A) Multi micro filtration unit for SEM sample preparation. An iso-pore membrane is placed under the removable funnel to collect bacteria. After sample preparation, the membrane is taken out of the unit. The membrane has a frame for placing it on the SEM specimen holder so that it can be easily inserted into the SEM specimen chamber. (B) Control (-) and antimicrobial treatment (+) were processed in parallel and sampled over time. (C) Bacterial samples were collected uniformly in a circular area 2 mm in diameter of the iso-pore membrane using a removable multi-well funnel of multi micro filtration unit.

SI Reference

1. W. S. Rasband, ImageJ, U. S. National Institutes of Health, Bethesda, Maryland, USA, https://imagej.nih.gov/ij/, 1997-2018.
